# O-40 - AN OVERVIEW OF THE UK HEALTH SECURITY AGENCY’S (UKHSA) HORIZON SCANNING PROCESS FOR ZOONOTIC INFLUENZAS (ZI)

**DOI:** 10.1093/pubmed/fdag046.036

**Published:** 2026-07-09

**Authors:** Miss Annabel Ridsdale, Dr Onyi Emefiene, Dr Liam Fitzpatrick, Dr Yingfen Hsia, Mr Varun Sathiyendra, Dr Sakib Rokadiya, Dr Angie Lackenby, Dr Richard Puleston, Dr Sophia Makki

**Affiliations:** UK Health Security Agency; UK Health Security Agency; UK Health Security Agency; UK Health Security Agency; UK Health Security Agency; UK Health Security Agency; UK Health Security Agency; UK Health Security Agency; UK Health Security Agency

## Abstract

**Background:**

Emerging respiratory infections are a major threat to global health security. The UKHSA national Acute Respiratory Infections team undertake horizon scanning (HS) as a proactive, systematic approach to identify emerging national and international respiratory threats to human health. Zoonotic Influenza (ZI) signals are routinely reviewed, risk assessed and escalated to ensure timely public health actions.

**Objectives:**

To describe the process for ZIs, highlighting pathways and outcomes.

**Methods:**

Respiratory HS involves assessing signals pertaining to respiratory pathogens predetermined by an expert body within a pathogen matrix. Signals enter the process externally e.g., World Health Organisation’s (WHO) Event Information Site or internally e.g., UKHSA syndromic surveillance. Using a structured risk framework, a weekly internal risk assessment determines whether the signal is downgraded, remains business-as-usual or is escalated to the UKHSA-wide HS process for further risk assessment. High priority signals with obvious concerning features are urgently escalated directly to Senior Officials.

**Results:**

Between January 2025 and March 2026, a total of eighty-two ZI signals were assessed (60% of all signals). Non-ZI signals included Legionella, Middle East Respiratory Syndrome and other high consequence infectious diseases. The majority of ZI signals were subtypes A(H5N1) (57%), A(H9N2) (14%) and A(H1N1v) (10%). Fifty-six signals involved confirmed human cases reported internationally. Thirty-two (40%) were escalated to the monthly HS process e.g., sporadic H9N2 cases in China. Three (10%) in 2025 were incorporated into existing Incident Management Teams. Previous signals have also undergone risk assessments and more detailed collaboration with external partners.

**Conclusions:**

This process ensures early detection, assessment and escalation of ZI threats with expert input feeding public health risk assessments, briefing notes, and collaboration with external experts such as WHO, underscoring its value in preparedness against pandemic threats. Future work includes considering how artificial intelligence can augment early risk assessment e.g., through early pathogen genomic analysis.

